# Development of an Arthroscopic Joint Capsule Injury Model in the Canine Shoulder

**DOI:** 10.1371/journal.pone.0147949

**Published:** 2016-01-25

**Authors:** David Kovacevic, Andrew R. Baker, Susan M. Staugaitis, Myung-Sun Kim, Eric T. Ricchetti, Kathleen A. Derwin

**Affiliations:** 1 Department of Orthopaedic Surgery, Cleveland Clinic, Cleveland, Ohio, United States of America; 2 Department of Biomedical Engineering and the Orthopaedic Research Center, Cleveland Clinic, Cleveland, Ohio, United States of America; 3 Department of Pathology, Cleveland Clinic, Cleveland, Ohio, United States of America; 4 Department of Orthopaedic Surgery, Chonnam National University College of Medicine, Gwangju, Republic of Korea; Mayo Clinic Minnesota, UNITED STATES

## Abstract

**Background:**

The natural history of rotator cuff tears can be unfavorable as patients develop fatty infiltration and muscle atrophy that is often associated with a loss of muscle strength and shoulder function. To facilitate study of possible biologic mechanisms involved in early degenerative changes to rotator cuff muscle and tendon tissues, the objective of this study was to develop a joint capsule injury model in the canine shoulder using arthroscopy.

**Methods:**

Arthroscopic surgical methods for performing a posterior joint capsulectomy in the canine shoulder were first defined in cadavers. Subsequently, one canine subject underwent bilateral shoulder joint capsulectomy using arthroscopy, arthroscopic surveillance at 2, 4 and 8 weeks, and gross and histologic examination of the joint at 10 weeks.

**Results:**

The canine subject was weight-bearing within eight hours after index and follow-up surgeries and had no significant soft tissue swelling of the shoulder girdle or gross lameness. Chronic synovitis and macroscopic and microscopic evidence of pathologic changes to the rotator cuff bony insertions, tendons, myotendinous junctions and muscles were observed.

**Conclusions:**

This study demonstrates feasibility and proof-of-concept for a joint capsule injury model in the canine shoulder. Future work is needed to define the observed pathologic changes and their role in the progression of rotator cuff disease. Ultimately, better understanding of the biologic mechanisms of early progression of rotator cuff disease may lead to clinical interventions to halt or slow this process and avoid the more advanced and often irreversible conditions of large tendon tears with muscle fatty atrophy.

## Introduction

Rotator cuff tears affect 40% or more of the population over age 60 and cost the US economy approximately $3 billion per year. The natural history of rotator cuff tears can be unfavorable as patients have been observed to develop significant fatty infiltration and muscle atrophy in the rotator cuff following the development of a tear [1], a condition coined “fatty atrophy”. Since muscle fatty atrophy is associated with a loss of muscle strength and shoulder function [2], and the process may be irreversible even following rotator cuff repair [3], there is a need to elucidate the etiology of this condition so that interventions for limiting or reversing fatty atrophy can be developed and used in the treatment of rotator cuff injury.

Clinical as well as animal studies have demonstrated that prolonged unloading or denervation is associated with fatty atrophy changes in the involved muscle. Four years after the initial diagnosis, non-operatively treated patients with massive rotator cuff tears were found to have significant progression of fatty atrophy and glenohumeral osteoarthritis [4]. Further, tendon release as well as muscle denervation led to the development of rotator cuff fatty atrophy in mouse, rat, rabbit, and sheep models by at least 6 weeks [5–10]. Adipocytes, intramuscular fat globules, and intramyocellular fat droplets were observed in the tenotomized and/or neurotomized muscles, which were associated with an increased expression of adipogenic and myogenic factors [7].

Inflammatory biochemical factors secondary to injury have also been implicated in the natural history of rotator cuff disease. It has been shown that levels of various matrix metalloproteinases (i.e., MMP-1, MMP-2, MMP-3, MMP-9, and MMP-13) were increased in synovial fluid of patients with rotator cuff tears as a function of tear size, and reflected as an increase in severity of synovial inflammation (i.e., IL-1β, IL-6, COX-2) and catabolic matrix breakdown [11–13]. These findings support the concept that the synovial tissue may respond to the insult of a rotator cuff tear by becoming hypertrophied and inflamed, and that a pro-inflammatory and catabolic state continues to worsen the severity of the tendon tear over time. Importantly, however, inflammatory biochemical changes in the injured shoulder joint have also been shown to be associated with degenerative collagen and vascular changes to adjacent *uninvolved* tendons [13], and fatty infiltration and muscular atrophy have been observed clinically in *uninvolved* rotator cuff muscles adjacent to the tear [14]. These findings suggest that alteration in inflammatory biochemical factors in an injured joint may contribute to degenerative changes in otherwise uninvolved and uninjured muscle and tendon tissues.

To facilitate the future study of possible biologic mechanisms involved in early degenerative changes to rotator cuff muscle and tendon tissues, an appropriate animal model is needed. As such, we propose that a joint capsule injury in the dog, where the capsule is distinct and separate from the overlying rotator cuff tendons [15], could introduce an isolated joint injury without muscle-tendon unloading or nerve injury. The beagle was chosen because they can be obtained at an advanced age (i.e., 9–10 years) that is more representative of the age of human patients (> 50 years) who present with degenerative rotator cuff pathology. We chose to create a joint capsule injury deep to the infraspinatus rather than the supraspinatus tendon because (a) the insertion of the joint capsule onto the humerus is well-delineated from the infraspinatus (but not the supraspinatus) tendon insertion, and (b) in future work where it may be desirable to create a concomitant tendon injury, compromising the supraspinatus tendon (but not the infraspinatus tendon) puts the animal at significantly increased risk of gross lameness (personal observation).

Therefore, the objective of this study is to develop a joint capsule injury model in the canine shoulder using arthroscopy, without frank injury to the overlying rotator cuff tendons. We propose use of an all arthroscopic technique in order to minimize the extent of soft tissue injury and the acute wound healing response compared to performing an open procedure. Although arthroscopy has commonly been used in the treatment of shoulder instability and forelimb lameness in dogs [16, 17], previous use in a research context is limited to investigating laser-assisted capsulorraphy in dogs [18]. We hypothesize that an isolated joint capsule injury model will be associated with pathologic changes in the shoulder joint. We anticipate that a joint capsule injury model can be used in future studies to investigate biochemical and pathologic changes to the shoulder joint and rotator cuff in the setting of a long-standing rotator cuff injury that does not involve tendon unloading or nerve damage.

## Materials and Methods

### Canine Shoulder Anatomy and Arthroscopic Portal Sites

**Fig 1:**
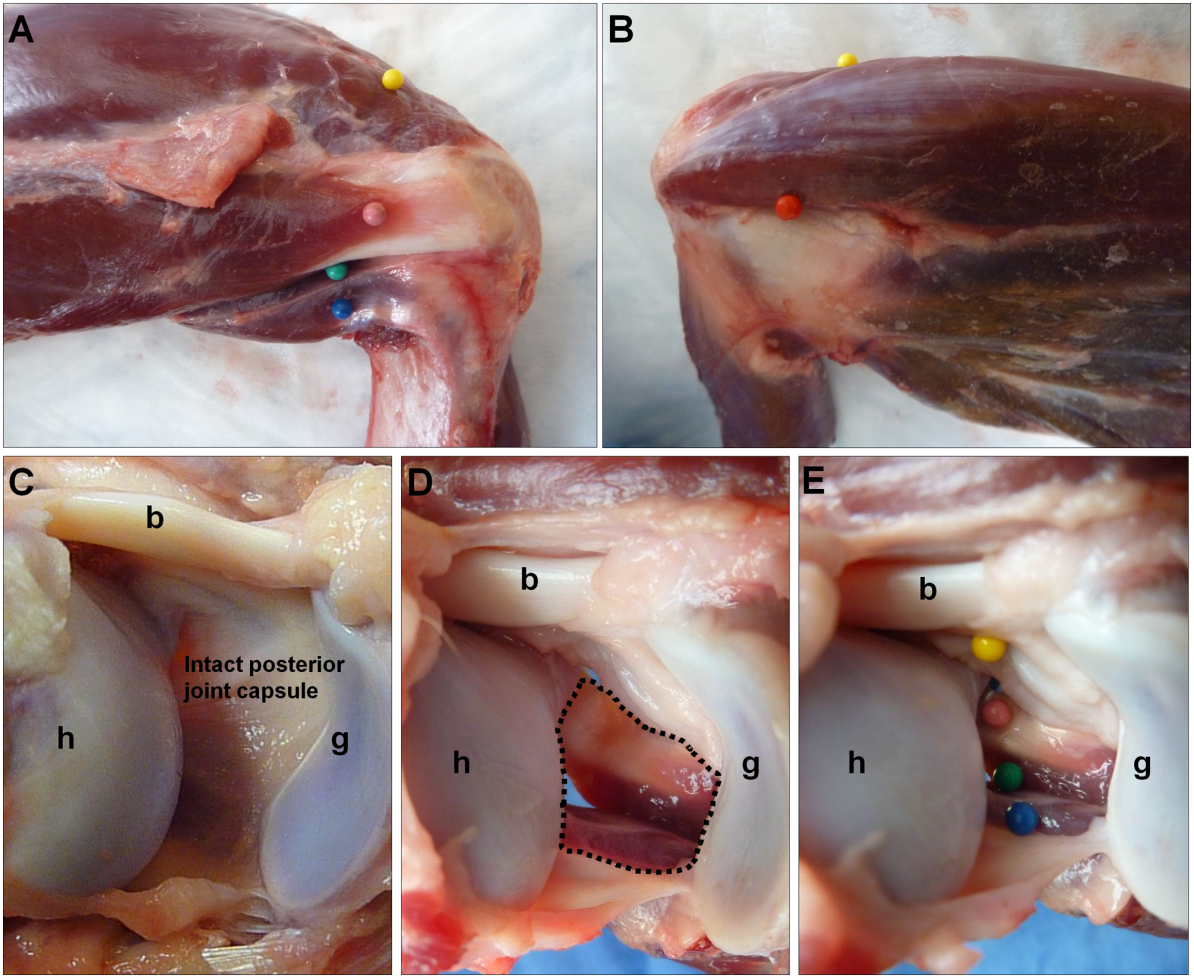
Cadaver canine shoulder (right) with skin and periscapular muscles removed. (A) Posterior bursal view: The supraspinatus (yellow), infraspinatus (pink) and teres minor muscles (blue) can be visualized. The posterior arthroscopic portal site (green) will be located between the infraspinatus and teres minor muscles. (B) Anterior bursal view: The anterior arthroscopic portal site (red) will be located between the supraspinatus and subscapularis muscles. (C)–(E) Posterior articular view (from viewing perspective of the anterior portal): Approximately 2 cm^2^ of the lateral joint capsule (C) under the infraspinatus will be resected from its humeral to glenoid insertions (D, dotted line), exposing the extra-articular rotator cuff muscles (E, colors as in Panel A) to the joint compartment. h = humeral head, g = glenoid, b = biceps tendon.

The gross anatomy for performing a posterior capsulectomy using arthroscopy, as well as two glenohumeral arthroscopic portals, were first defined in cadaveric canine shoulders which were available from an unrelated study (mongrels, 22–25 kg; Marshall BioResources, North Rose, NY). We note that the anatomic descriptors (anterior/posterior or medial/lateral) used throughout this manuscript follow the conventions used for the human shoulder. The overlying skin and superficial periscapular muscles were carefully dissected to reveal the rotator cuff muscles. The supraspinatus, infraspinatus and teres minor muscles can be appreciated from a posterior view of the shoulder (Fig 1A). We determined that the posterior arthroscopic portal site will be located between the infraspinatus and teres minor muscles (green marker, Fig 1A), and the anterior arthroscopic portal site will be located between the supraspinatus and subscapularis muscles (red marker, Fig 1B). Further, we determined that approximately 2 cm^2^ of the posterior joint capsule under the infraspinatus (Fig 1C) will be resected from its humeral to glenoid insertions (Fig 1D), exposing the extra-articular rotator cuff muscles (Fig 1E) to the intra-articular space. We note that the following critical structures should be avoided: humeral head articular cartilage, glenoid articular cartilage, and the biceps and subscapularis tendons.

### Arthroscopic Shoulder Capsulectomy

**Fig 2:**
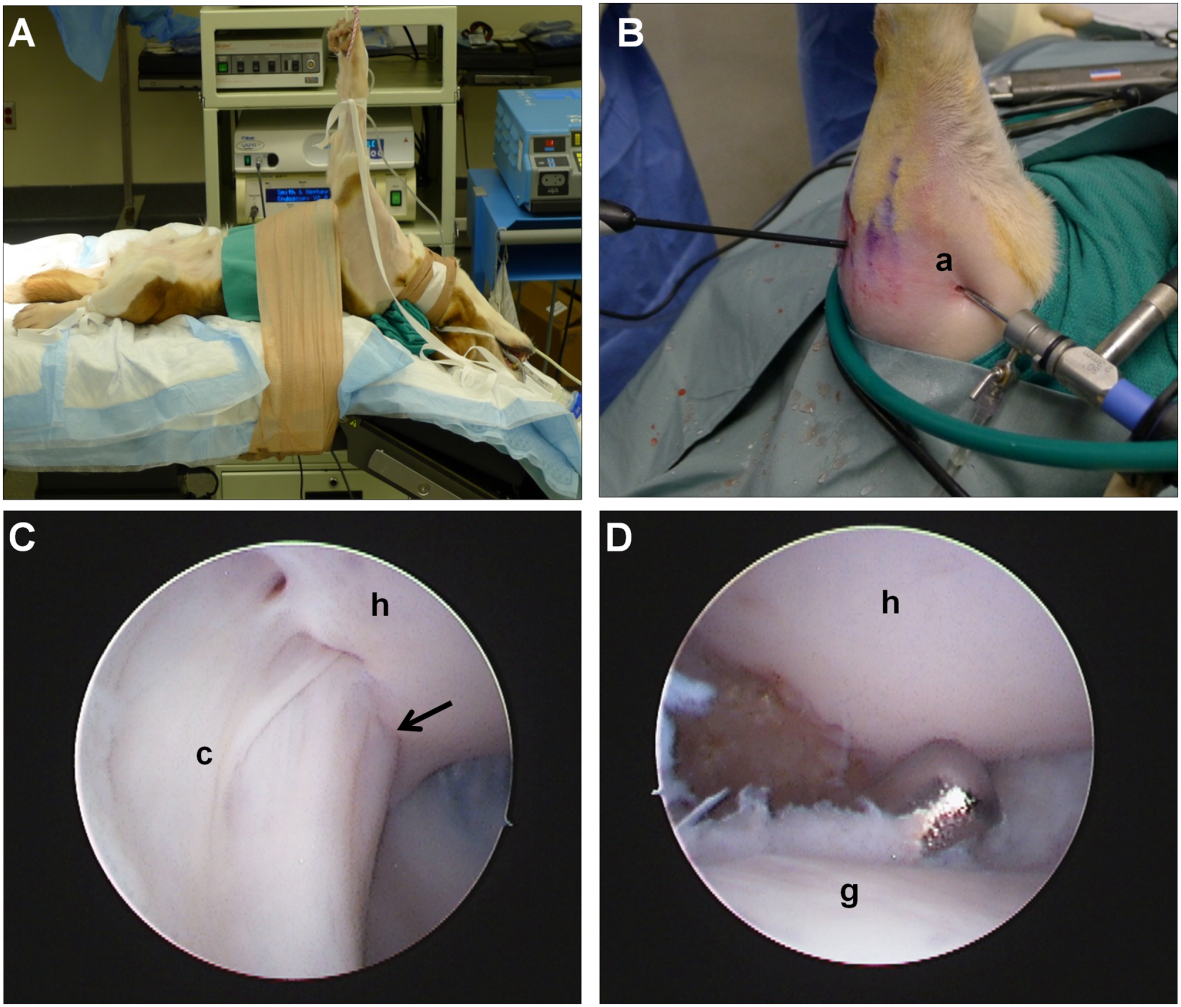
Arthroscopic shoulder capsulectomy. (A) The animal was placed in a dorsal recumbent position with neck extension, and a forelimb suspension system was utilized to provide forelimb traction and ensure adequate glenohumeral joint distraction. (B) Arthroscopic posterior capsulectomy was performed with instrumentation in the posterior portal and the arthroscope in the anterior portal. (C) The humeral insertion of the intact posterior capsule visualized from the anterior portal (photo taken in a cadaver shoulder for clarity). The arrow denotes the insertion of the capsule at the supraspinatus/infraspinatus transition. (D) Removal of any remaining capsular tissue from the humeral or glenoid ends of the defect was done with an arthroscopic shaver (photo taken in a cadaver shoulder for clarity). a = anterior portal, c = joint capsule, h = humeral head articular surface, g = glenoid articular surface.

This study was approved by the IACUC at the Cleveland Clinic (#2011–0549). Procedures were first developed on eight canine cadaveric shoulders and then performed bilaterally on a living canine subject. Specifically, one retired female breeder beagle (9 years old, 12 kg, Ridglan Farms, Mt. Horeb, WI) received a transdermal Fentanyl patch (50μg/hr) 18 hours prior to surgery. Just prior to anesthetic induction, a 0.1 mg/kg subcutaneous injection of acepromazine in the nape of the neck and an oral dose of 0.2 mg/kg meloxicam for analgesia were administered. The dog was then anesthetized with an intravenous dose of sodium methohexital (10 mg/kg) to effect and then intubated orotracheally and maintained on isoflurane in oxygen (3%). The dog received a single dose of intravenous cefazolin (20 mg/kg) as a prophylactic antibiotic prior to surgery. The animal was placed in a dorsal recumbent position with neck extension, and a forelimb suspension system was utilized to provide forelimb traction and ensure adequate glenohumeral joint distraction (Fig 2A). Additional clearance around the head and neck was created by downward tilt of the surgical table. Both forelimbs were prepped and draped in the usual sterile manner.

Arthroscopic capsulectomy was performed on each shoulder in sequence, and intra-operative pain levels were continuously assessed by monitoring for increased heart rate, blood pressure, and reflex response to stimulus. First, posterior and anterior skin only incisions (3–5 mm) were made with a scalpel. Using a conical obturator, the posterior portal was established between the infraspinatus and teres minor tendons and a cannulated arthroscopic sheath was placed. A 30°, 2.3 mm arthroscope (Stryker Endoscopy, San Jose, CA) was inserted into the cannula to visualize the triangulated anterior compartment structures-biceps tendon, medial glenohumeral ligament, and subscapularis tendon. Using an outside-in technique with arthroscopic visualization, a spinal needle and then K-wire were inserted into this triangular space to establish the anterior portal. The arthroscope in the posterior portal was replaced with a switching stick and moved to the anterior portal, permitting visualization of the posterior compartment structures, namely the glenoid labrum, lateral glenohumeral ligament, and posterior joint capsule. Joint distention was maintained using lactated Ringers solution simply on a gravity feed, so no egress cannula was necessary. A combination of a 15° tissue elevator (Arthrex, Naples, FL), banana knife (Arthrex), 3.5 mm 90°and 2.5 mm 30°suction electrodes (DePuy Mitek, Raynham, MA), and a 3.5 mm shaver with a full radius Incisor^®^ blade (Smith & Nephew, Andover, MA) were alternatingly introduced through the posterior portal to perform the posterior joint capsulectomy with visualization from the anterior portal (Fig 2B).

**Fig 3:**
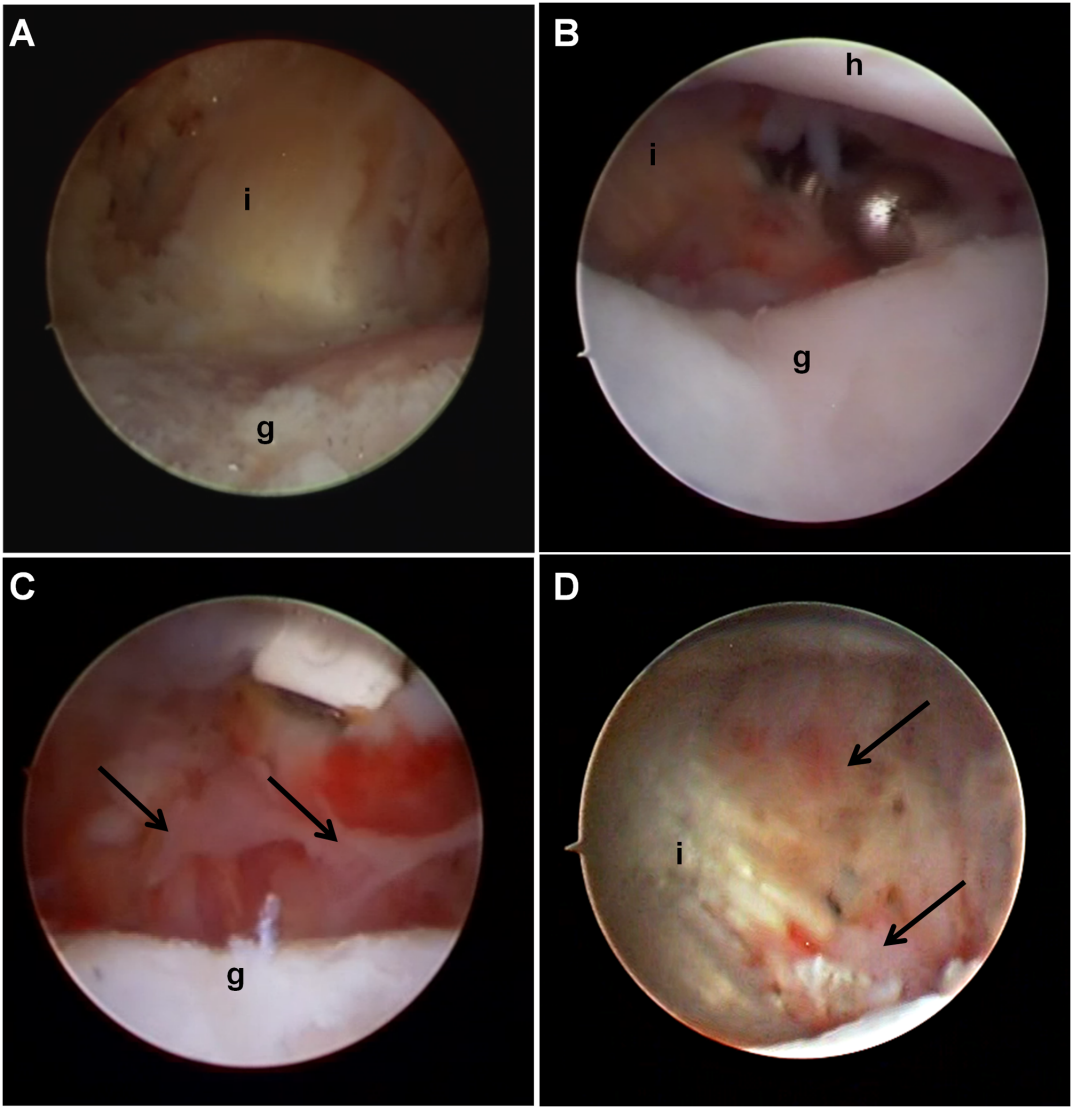
Creation and surveillance of a posterior joint capsule injury, representative photographs from the left shoulder. (A) At index procedure, completion of the capsulectomy exposed the deep surface of the infraspinatus tendon and muscle, (B) At two weeks, there was negligible healing tissue present at the capsulectomy site. (C) At four weeks, the capsulectomy site appeared hemorrhagic and white, and thin fibrous tissue (arrows) was present. (B) At eight weeks, the deep surface of the infraspinatus tendon appeared yellow with fibrous tissue (arrows) originating medial to the glenoid rim and extending laterally to the deep surface of the tendon. i = infraspinatus tendon, h = humeral head articular surface, g = glenoid articular surface.

With the posterior joint capsule visualized (Fig 2C), the tissue elevator was first used to detach the glenoid labrum from the posterior glenoid rim within the boundaries of the proposed capsulectomy. (Since there is no clear distinction between the labrum and capsule insertion along the posterior glenoid rim, it is technically easier to detach the labrum together with the capsule). With the labrum detached, the banana knife and suction electrodes were then used to incise the inferior and superior edges of the capsule defect. The inferior boundary of the capsule defect extended from the humeral head to the glenoid rim along a line directly adjacent to the posterior portal (Fig 1D). The superior boundary of the capsule defect extended from the humeral insertion of the capsule at the supraspinatus-infraspinatus transition to the glenoid rim (Fig 1D). Finally, a mechanical shaver was introduced to remove any remaining capsular tissue on the humeral head or glenoid rim (Fig 2D). The capsular defect was approximately 1 cm (anterior-posterior) by 2 cm (medial-lateral) in size. Completion of the capsulectomy exposed the deep surface of the infraspinatus tendon laterally and the myotendinous junction medially (Figs 1E and 3A). Since a gravity feed of lactated Ringer’s was maintained throughout the procedure, joint lavage was constant and no additional lavage was performed after the procedure. The arthroscopic tools and camera were then removed from the joint, and the skin incisions were closed with one or two 3–0 nylon simple sutures (Ethicon, Inc., Somerville, NJ).

Postoperatively, no rescue analgesia was required. Following each procedure, the dog was continually monitored by veterinary staff until regaining sternal recumbence. Once achieved, the dog was checked hourly until weight bearing and at least twice daily thereafter. An additional oral dose of 0.1 mg/kg meloxicam was given for analgesia the morning after surgery, and the Fentanyl patch (50 μg/hr) was maintained for 72 hours after surgery. Dogs were housed in standard cages (95”x33”x65”) with raised grated floors and fed 25 g/kg Harlan Teklad Canine Diet 8653 (Harlan Industries, Indianapolis, IN) daily. As per standard of care the dog was allowed immediate, unrestricted cage activity, unrestricted activity in the dog housing room during daily cage cleaning, cage toys and routine enrichment activities.

### Surveillance Shoulder Arthroscopy

The beagle underwent bilateral surveillance glenohumeral arthroscopy at two, four, and eight weeks following the index procedure. These follow up procedures were performed to visualize the healing environment of the glenohumeral joint as well as mechanically debride any healing tissue that may have been present at the capsulectomy site. A diagnostic evaluation of the anterior compartment was performed using the posterior portal, and evaluation of the posterior compartment was performed using the anterior portal. The surgical technique for portal establishment, evaluation, and debridement was performed as described above. Post-operative activity and pain-management was as described above.

### Necropsy and Tissue Procurement

The beagle was euthanatized at ten weeks following the index procedure using a lethal IV injection of barbiturate (1 mL/4.5 kg; Beuthanasia-D^®^; WA Butler, Dublin, OH). Approximately 1–2 ml of synovial fluid was able to be collected from the open joint at necropsy. A careful dissection of both shoulders was performed. The infraspinatus tendon, including its humeral insertion, the biceps tendon including its glenoid insertion, and a cross-sectional slice of the infraspinatus tendon/muscle adjacent to the joint capsule defect were recovered from each shoulder. The same tissue samples were also recovered from one age-matched normal beagle shoulder. The specimens from the tendinous insertions were fixed in 10% neutral buffered formalin (Sigma-Aldrich, St. Louis, MO) for 48 hours, decalcified in 10% EDTA (Sigma-Aldrich) for 3–5 days, processed into paraffin blocks and sectioned and stained with hematoxylin and eosin (H&E). The muscle specimens were flash frozen in isopentane cooled in liquid nitrogen, and stored at -80°C until sectioning and staining with H&E. All of the histologic sections were reviewed and described by one of the study co-authors who is a board certified pathologist (SMS).

## Results

### Postoperative Clinical Observations

The dog’s post-operative heart and respiratory rates were 80–120/min and 20–35/min respectively, and there was no evidence the animal experienced any undue distress from surgery. The dog was weight-bearing within eight hours after the index and all follow-up surgeries. Moderate fluid extravasation in the shoulder girdle and extending to the lower limb was observed, which completely resolved within 48 hours of each surgery. The dog did experience moderate difficulty lying down and rising to a standing position for 1–2 days following each of the index or follow-up procedures, but otherwise no gross lameness was observed. The dog otherwise had no complications, and no significant soft tissue swelling of the shoulder girdle was observed. The analgesic protocol of transdermal fentanyl augmented with two doses of oral meloxicam appeared to be sufficient for pain management in this model.

### Surveillance Shoulder Arthroscopy

Arthroscopic surveillance observations at two, four, and eight weeks were similar for both shoulders. At two weeks, the tissue near the anterior portal was hyperemic, grossly consistent with an acute synovitis. Wispy strands of fibrous tissue were present at the glenoid rim and humeral head of the capsulectomy site, and these fibrous strands were removed with a mechanical shaver (Fig 3B). At four weeks, there was considerable synovitis throughout the glenohumeral joint. Villous synovial hyperplasia was noted near the anterior portal, the biceps root was stained with hemosiderin, and the remaining capsule appeared hyperemic. Thin, wispy fibrous tissue was observed on the deep surface of the infraspinatus tendon, which was removed with a shaver (Fig 3C). At eight weeks, synovitis persisted throughout the joint. Although there was no tissue in continuity spanning the gap between the humeral head and glenoid rim at the site of the capsulectomy, fibrovascular tissue extended from the glenoid onto the deep surface of the infraspinatus muscle-tendon unit, indicating partial healing at the capsulectomy site (Fig 3D).

### Necropsy Findings

**Fig 4:**
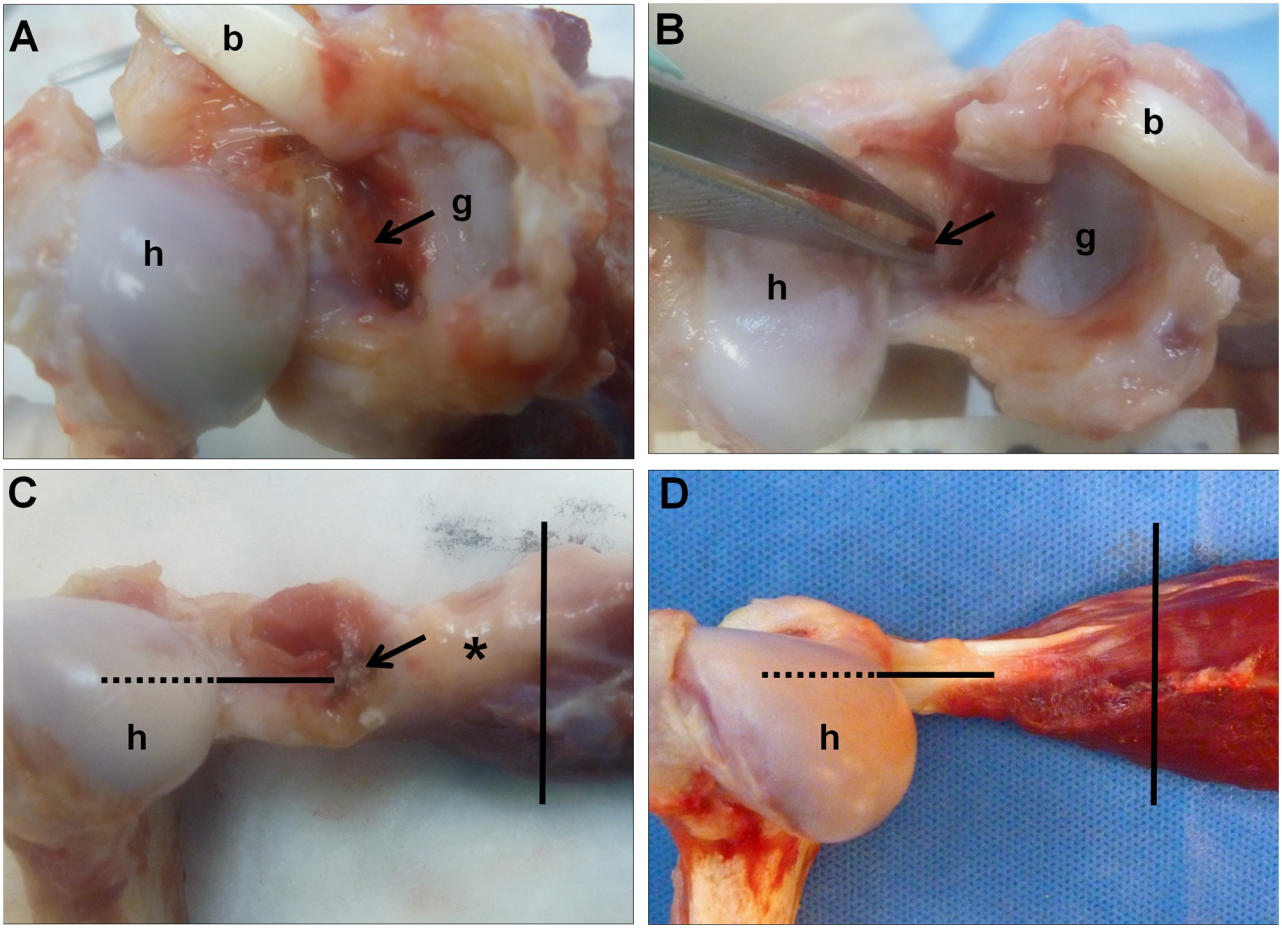
Necropsy findings at 10 weeks after the index procedure, representative images from the right shoulder. (A) Fibrovascular plaques were observed over the deep surface of the infraspinatus tendon (arrow), extending to the glenoid rim at the level of the scapular neck. (B) This material was thin, friable, and discontinuous (arrow), demonstrating that the capsular injury had remained patent. (C) The lateral infraspinatus muscle was edematous and its deep surface was covered with fibrovascular plaques laterally (arrow) and fibro-fatty tissue that encapsulated an accumulation of fluid medially (asterisk). (D) The deep surface of a non-operated normal canine infraspinatus tendon/muscle is shown for comparison. h = humeral head, g = glenoid, b = biceps tendon. Lines on C and D denote location of histologic sections of infraspinatus tendon (horizontal) and infraspinatus muscle (vertical).

Gross observations at necropsy (10 weeks) were similar for both shoulders. There was no obvious effusion. The biceps tendons were thickened and yellow in color suggestive of hemosiderin deposits, and the biceps roots at the glenoid rim were thickened with plaque-like tissue. Fibrovascular plaque-like tissue was also observed over the deep surface of the infraspinatus tendons, extending to the glenoid at the level of the scapular neck (Fig 4A). This tissue was thin, friable, and discontinuous, demonstrating partial healing but also that the capsular injury had remained patent in both shoulders (Fig 4B). An accumulation of fluid was present deep to the infraspinatus muscle and adjacent to the infraspinatus fossa and extending approximately 1.5 cm medial to the scapular notch. The lateral infraspinatus muscle was edematous and its deep surface was covered with fibrovascular plaques laterally and fibro-fatty tissue that encapsulated a portion of a fluid-filled cyst medially (Fig 4C). The humeral and glenoid articular surfaces appeared smooth and uninjured (Fig 4A–4C). For comparison, the normal canine infraspinatus tendon/muscle is shown in Fig 4D.

### Histologic Observations

#### Biceps Tendon and its Insertion to Glenoid

**Fig 5:**
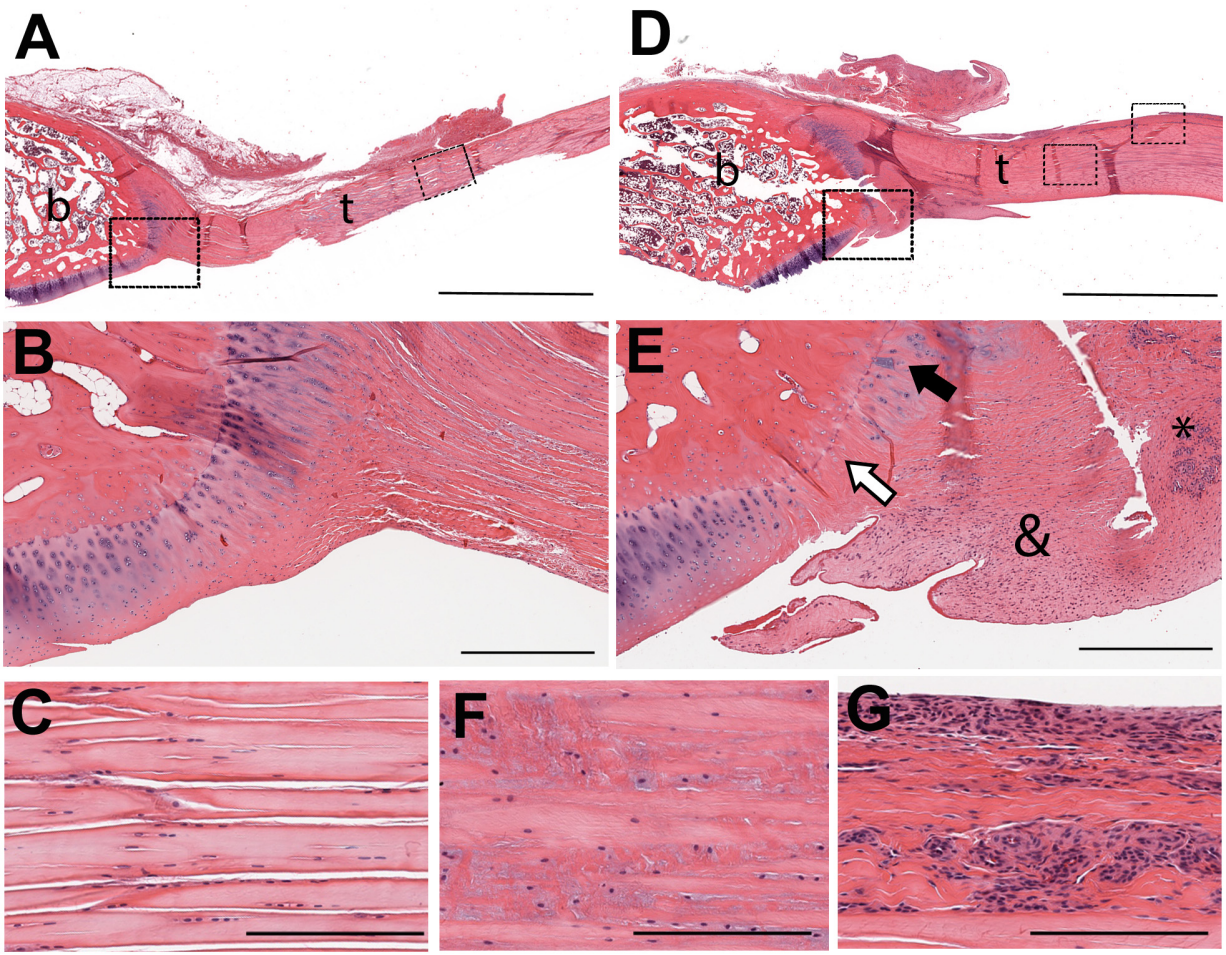
Biceps tendon and glenoid insertion. Longitudinal histologic sections from non-operated normal (A-C) and operated (D-G) animals (t = tendon; b = bone). The insertion of the non-operated animal showed the characteristic fibrocartilaginous transition zone with a well-defined tidemark and aligned chondrocytes (B, where B is a higher magnification of the region denoted by the left box on A). The adjacent tendon was characterized by parallel rows of tenocytes sandwiched between highly aligned collagen fibers (C, where C is a higher magnification of the region denoted by the right box on A). Ten weeks after posterior arthroscopic joint capsule injury, the tendon and its glenoid insertion showed reactive/reparative changes (D-G). The tendon in the operated animal (D) was thicker than normal (A). The distribution of chondrocytes at the tidemark was not uniform (E, where E is a higher magnification of the region denoted by the left box on D), and some foci showed few or no chondrocytes (white arrow) while others showed clustering of chondrocytes (black arrow), suggestive of proliferation. The tendon adjacent to the insertion showed villous hyperplasia (ampersand) and foci of marked hypervascularity (asterisk) (E). The rest of the tendon showed increased numbers of cells surrounded by basophilic ground substance (F, where F is a higher magnification of the region denoted by the middle box on D). There was marked hypervascularity along the entire length of the superior aspect of the tendon (G, where G is a higher magnification of the region denoted by the right box on D). Hematoxylin and eosin stain. Scale bar = 5mm (A, D), 500 microns (B, E), 200 microns (C, F, G).

Longitudinal histologic sections of the biceps tendon were taken in a plane that included its glenoid insertion. In the non-operated normal animal (Fig 5A–5C), the biceps-glenoid insertion showed the characteristic fibrocartilaginous transition zone with a well-defined tidemark and chondrocytes aligned in perpendicular columns (Fig 5B). The adjacent tendon was characterized by parallel rows of tenocytes sandwiched between highly aligned collagen fibers (Fig 5C). Ten weeks after posterior arthroscopic joint capsule injury (Fig 5D–5G), the biceps tendon was thicker than normal (Fig 5D). At the biceps-glenoid insertion, the distribution of the chondrocytes at the tidemark was not uniform. Some foci showed few or no chondrocytes (white arrow) while others showed clustering of chondrocytes, suggestive of proliferation (black arrow) (Fig 5E). The tendon adjacent to the insertion showed villous hyperplasia (ampersand) and foci of marked hypervascularity (asterisk) (Fig 5E). The rest of the tendon showed increased numbers of cells surrounded by basophilic ground substance (Fig 5F). There was marked hypervascularity along the entire length of the superior aspect of the tendon (Fig 5G). The inferior aspect distal to the insertion was normal in appearance (not shown).

#### Infraspinatus Tendon and its Insertion to Humerus

**Fig 6:**
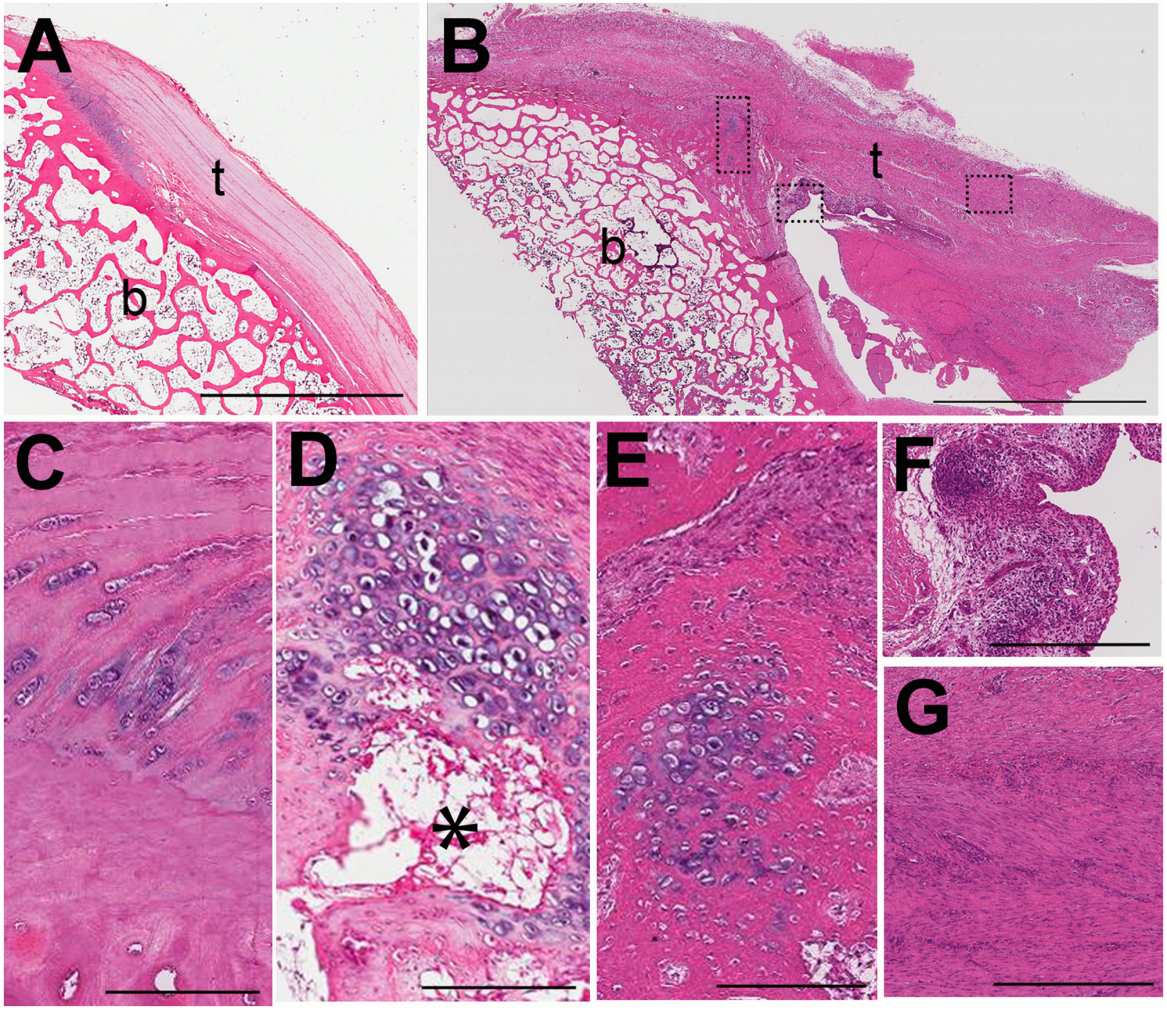
Infraspinatus tendon and humeral insertion. Longitudinal histologic sections from non-operated normal (A) and operated (B-G) animals (t = tendon; b = bone). At low magnification, the infraspinatus tendons in the operated shoulders (B) were markedly thicker than normal (A). Examination of all sections revealed a wide degree of pathologic changes at the tendinous insertion site, including irregularly distributed and clustered chondrocytes (C), marked proliferation and clustering of chondrocytes separated from the tidemark by vascular invasion (asterisk) (D), and deposition of osteoid around clusters of chondrocytes consistent with endochondral bone formation (E, where E is a higher magnification of the region denoted by the left box on B). The infraspinatus tendons adjacent to their insertion sites were markedly hypercellular consisting of stromal cells and a chronic inflammatory infiltrate with increased vascularity (F, where F is a higher magnification of the region denoted by the middle box on B). The operated tendons more medial to the insertion sites show an increased number of hypertrophied tenocytes and diffusely distributed lymphocytes (G, where G is a higher magnification of the region denoted by the right box on B). Hematoxylin and eosin stain. Scale bar = 5 mm (A, B); 200 microns (C-E); 500 microns (F, G).

The histology of the infraspinatus tendons on longitudinal sections in the non-operated normal animal (Fig 6A) is similar to that observed for the normal biceps tendon (Fig 5A); however, in the operated animal the infraspinatus tendons showed a greater degree of pathology than the biceps tendons. At low magnification, the operated infraspinatus tendons were markedly thicker than normal in all sections examined (Fig 6B). The histology of the infraspinatus insertion to humerus showed a great degree of variability. Examination of all sections reviewed at high magnification from both operated shoulders revealed features that suggest a histologic progression of pathologic bone formation. Fig 6C from the right shoulder of the operated animal had features similar to that seen at the biceps tendon insertion in the operated shoulder, namely, chondrocytes were irregularly distributed and frequently clustered. Fig 6D is from the left shoulder at a level that is superior to that shown in Fig 6B. Here, there was a marked proliferation and clustering of chondrocytes that have larger lacunae and are surrounded by basophilic ground substance, similar to hyaline cartilage. This focus was separated from the location of the tidemark by what appeared to be vascular invasion (asterisk), similar to what has been described for early stages of endophyte formation in the Achilles tendon [19]. The histology of the region in Fig 6E showed deposition of osteoid around clusters of chondrocytes consistent with endochondral bone formation. The operated infraspinatus tendons adjacent to their insertion sites were markedly hypercellular. In addition to a proliferation of stromal cells, there was also a chronic inflammatory infiltrate and increased vascularity (Fig 6F). Tendon more medial to the insertion site showed an increased number of hypertrophied tenocyte nuclei (Fig 6G). Cells consistent with lymphocytes were also present but diffusely distributed (Fig 6G).

#### Infraspinatus Muscle

**Fig 7:**
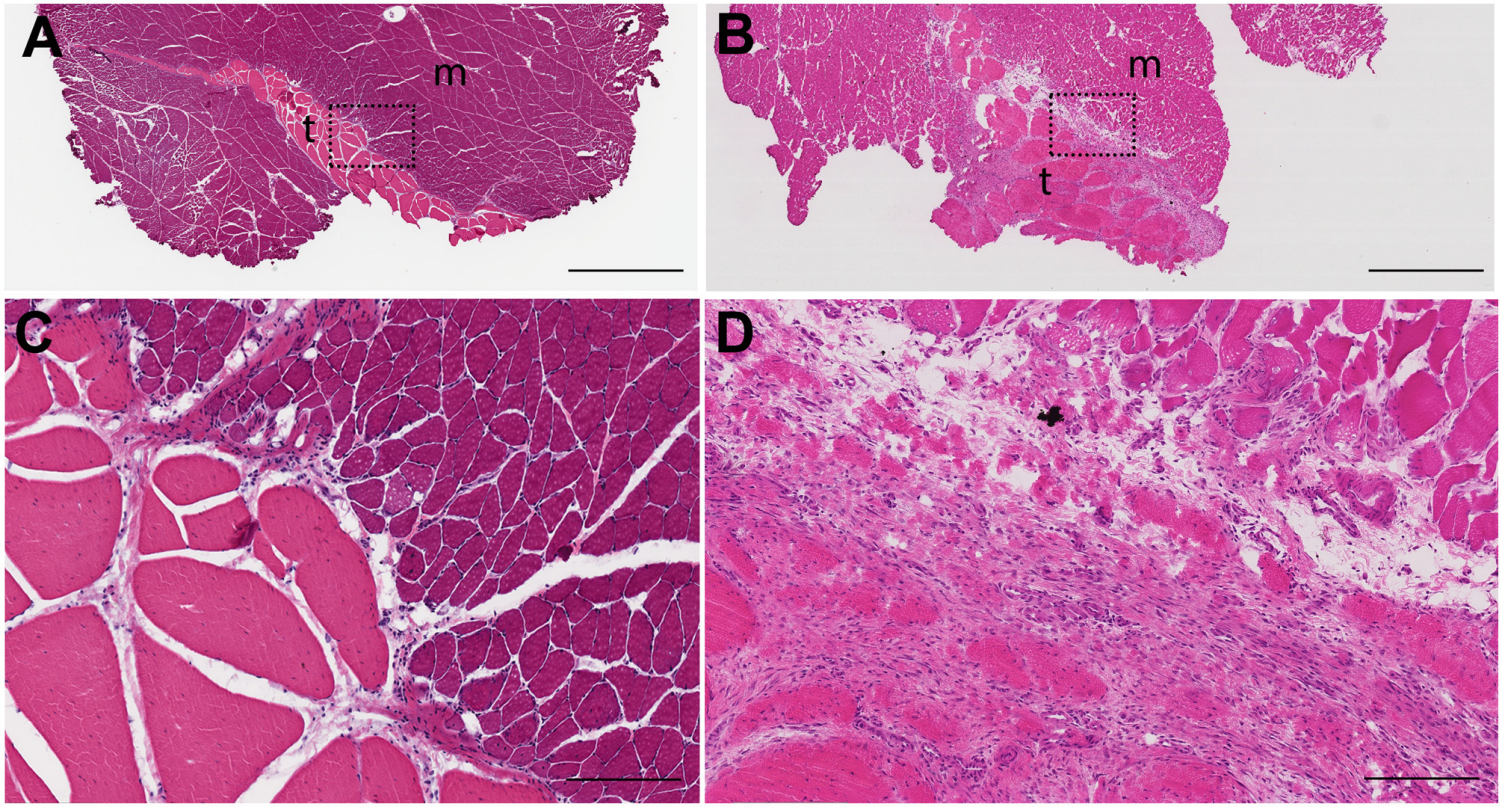
Infraspinatus muscle. The muscle-tendon interface of the infraspinatus muscle ten weeks post joint capsulectomy showed reactive/reparative changes. Low magnification cross-sections of regions sampled from non-operated normal (A) and operated (B) animals (t = tendon; m = muscle). Boxes delineate regions shown at high magnification in C and D respectively. In contrast to the normal discrete muscle-tendinous interface (C), the interface in the operated animals was disorganized and characterized by a marked increase in stromal cells and capillaries, and small, rounded muscle cells (D). Hematoxylin and eosin stain. Scale bar = 2 mm (A, B); 200 microns (C, D).

The infraspinatus muscles from non-operated normal (Fig 7A) and joint capsulectomy operated (Fig 7B) conditions were sampled for cross-sectional histologic examination at the level of the tendinous insertion (Fig 4D). High magnification of the non-operated normal specimen showed a discrete muscle-tendon interface (Fig 7C). The tendon fascicles were of low cellularity. The endotenon septa were narrow, and the capillaries within them were inconspicuous. The muscle fibers immediately adjacent to the tendon showed the expected variability in size, angulated morphology, and multiple centrally-located nuclei. Muscle fibers more distal to the tendon insertion were uniform in size, and had a polygonal morphology and peripherally located nuclei. Ten weeks after posterior arthroscopic joint capsule injury, the muscle-tendon interface appeared disorganized and both the tendon and muscle at the interface showed reactive changes (Fig 7D). The tendon fascicles were much wider than normal due to a marked increase in stromal cells and capillaries. These changes are suggestive of an active reparative process. Most of the muscle fibers at this interface were small and rounded. This morphology is characteristic of myopathic atrophy. The muscle fibers approximately 1 mm away from the deep muscle-tendon interface and in the entire superficial portion of the muscle showed a normal morphology (not shown).

## Discussion

Muscle atrophy in the absence of tendon unloading or nerve injury has been observed in intact rotator cuff muscles adjacent to injured tendons [20–22]. These observations suggest that other factors in an injured joint may contribute to degenerative changes, and our objective was to develop an animal model where the role of joint capsule injury in precipitating degenerative changes to uninvolved muscle and tendon tissues could be investigated. We used an arthroscopic technique in order to minimize the extent of soft tissue injury and the acute wound healing response compared to performing an open procedure to create the injury. The arthroscopic model would also allow the investigator to assess and observe soft tissue injury and repair mechanisms at serial time points within the same animal, with the added benefit of potentially procuring biopsy samples at specified time points [23].

We were able to create an isolated joint capsulectomy in the canine shoulder using an arthroscopic technique with two portals (anterior and posterior). The ~1 x 2 cm defect extended from the humeral insertion of the capsule at the supraspinatus-infraspinatus transition superiorly to the infraspinatus-teres minor boundary inferiorly, and from the humerus laterally to the glenoid rim medially. We were able to verify the reproducibility of our approach by first performing the procedures in eight canine cadaveric shoulders before transitioning to a living canine subject. In one canine subject where a joint capsulectomy was created and maintained bilaterally by arthroscopy for 10 weeks, no complications, significant soft tissue swelling of the shoulder girdle, or gross lameness were observed.

In support of our study hypothesis, the isolated joint capsule injury model showed pathologic changes in the shoulder joint in the absence of any apparent tendon unloading or nerve damage. Macroscopically, there was synovitis, tendon thickening and fibrovascular tissue deposition suggesting that the shoulder was in a chronic inflammatory/reparative state throughout the 10 week study period. At necropsy, we documented a spectrum of histologic changes at the tendon-bone insertion sites and in the tendon, myotendinous junction and adjacent muscle to support the interpretation of an ongoing reparative response in all rotator cuff tissues. Since the joint capsule is distinctly separate from the rotator cuff tendons in the canine, we are confident that mechanical tissue injury from the surgical procedures was limited to the joint capsulectomy and perhaps the supraspinatus and teres minor muscles adjacent to the arthroscopic portals. Hence it is reasonable to conclude that the chronic injury response observed in the synovium and infraspinatus and biceps tendons and muscles stems from biochemical and/or mechanical changes in the joint secondary to the injury model. However, the extent to which this injury condition arises from the persistent joint capsule injury or provocation by repeated surveillance arthroscopy cannot be determined by this study. It is also not known if the pathologic changes observed would progress in time to the more degenerative pathology observed in the torn human rotator cuff [24].

The canine joint capsulectomy model is intended to capture the chronicity and intra-articular nature of rotator cuff injury in the human condition. Nevertheless, it is important to acknowledge that, in contrast to human patients whose chronic rotator cuff tears are degenerative in nature secondary to repetitive activity, microtrauma, impingement, and/or aging, and have limited intrinsic healing capacity [25], a robust healing response was initiated following the surgically induced capsulectomy in the canine model. Repeated arthroscopic interventions were prescribed in the experimental design of this study to ensure that a persistent (open) capsule defect was maintained. It is unknown whether the capsulectomy would have effectively closed secondary to normal physiological reparative responses in the absence of arthroscopic surveillance and debridement. It also is possible that simply incising the capsule (i.e., capsulotomy) would have resulted in less trauma, less bleeding, and possibly less healing compared to capsular excision, and suggest that a capsulotomy injury model should be explored in future work. Furthermore, it should be noted that dogs do not develop muscle atrophy and fatty accumulation to the same extent as humans [26], making them perhaps less sensitive as a model for studying myopathic pathology. Despite these inherent limitations of animal models (artificial injury mechanism, robust healing, less pathology), we believe the canine model of arthroscopic shoulder injury presented herein has the potential to allow investigators to study the role of joint capsule injury, tendon/muscle unloading, and nerve injury—in isolation or combination—on the development of rotator cuff pathologies.

The data reported here are intended to demonstrate technical feasibility and proof-of-concept for an isolated joint capsule injury model in the canine. This preliminary data enabled us to identify key variables to be investigated for refinement of the model. In addition to evaluation of more animals and sham surgeries, additional groups that do not undergo arthroscopic surveillance, or that undergo surveillance with synovial fluid sampling and tissue biopsy, are indicated. Future work should utilize, a 70°, 2.3 mm arthroscope, which was not available to us for this study but would have allowed better visualization of the structures medial to the glenoid rim so as to assess the scapular neck, which is where we believed the healing tissue on the deep surface of infraspinatus tendon originated from based on the necropsy findings.

## Conclusion

In summary, we have developed a new animal model of isolated joint capsule injury in the canine shoulder. We report on the technical methods for creating an injury to the shoulder joint capsule using an arthroscopic technique, demonstrate that the arthroscopic injury can be made without introducing gross lameness to the animal, and show that chronic synovitis and macroscopic and microscopic evidence of pathologic changes to the rotator cuff bony insertions, tendons, myotendinous junctions and muscles occur. Future work is needed to further define the extent of pathologic changes and their progression in rotator cuff disease. Ultimately, a better understanding of the mechanisms of early progression of rotator cuff disease may lead to clinical interventions to halt or slow this process and avoid more advanced and often irreversible conditions of tendon tearing and muscle unloading leading to pain, fatty atrophy and loss of shoulder function.
